# Algebraic properties of the maps $\chi _n$

**DOI:** 10.1007/s10623-024-01395-w

**Published:** 2024-04-10

**Authors:** Jan Schoone, Joan Daemen

**Affiliations:** Digital Security, Radboud University, Nijmegen, The Netherlands

**Keywords:** Boolean maps, Chi, Cryptography, Polynomial maps, Symmetric cryptography, 94D10, 12E20, 14R15, 14R99

## Abstract

The Boolean map $\chi _n :\mathbb {F}_2^n \rightarrow \mathbb {F}_2^n,\ x \mapsto y$ defined by $y_i = x_i + (x_{i+1}+1)x_{i+2}$ (where $i\in \mathbb {Z}/n\mathbb {Z}$) is used in various permutations that are part of cryptographic schemes, e.g., Keccak-f (the SHA-3-permutation), ASCON (the winner of the NIST Lightweight competition), Xoodoo, Rasta and Subterranean (2.0). In this paper, we study various algebraic properties of this map. We consider $\chi _n$ (through vectorial isomorphism) as a univariate polynomial. We show that it is a power function if and only if $n=1,3$. We furthermore compute bounds on the sparsity and degree of these univariate polynomials, and the number of different univariate representations. Secondly, we compute the number of monomials of given degree in the inverse of $\chi _n$ (if it exists). This number coincides with binomial coefficients. Lastly, we consider $\chi _n$ as a polynomial map, to study whether the same rule ($y_i = x_i + (x_{i+1}+1)x_{i+2}$) gives a bijection on field extensions of $\mathbb {F}_2$. We show that this is not the case for extensions whose degree is divisible by two or three. Based on these results, we conjecture that this rule does not give a bijection on any extension field of $\mathbb {F}_2$.

## Introduction

In this paper, we consider the Boolean maps $\chi _n :\mathbb {F}_2^n \rightarrow \mathbb {F}_2^n,\ x \mapsto y$ that are defined by $y_i = x_i + (x_{i+1}+1)x_{i+2}$, with $i\in \mathbb {Z}/n\mathbb {Z}$. For $n=5$, it is used in Keccak-f [2] (which is part of the NIST standard SHA-3 [22]) and ASCON [14] (the winner of the NIST lightweight competition [23]). For $n=3$, it is used in Xoodoo [10]. Rasta [13] uses $\chi _n$ where *n* is the block-length (*n* is always odd). Lastly, Subterranean (2.0) ([7] and [11]) uses $\chi _{257}$.

We know, from [8], that $\chi _n$ is invertible if and only if *n* is odd. Recently, from [20], we know a direct formula for $\chi _n^{-1}$. The order of $\chi _n$, and its cycle structure, are also known, see [30].

As $\chi _n$ is used in so many cryptographic applications, it is important to understand these maps very well. Each of the properties of $\chi _n$ could be exploited in an attack, or conversely be used to argue for security properties. For instance, in [8] and [9], the differential and correlation properties (related to differential [3] and linear [21] cryptanalysis) have been studied.

In this paper, we study some of the algebraic properties. E.g., the map $\chi _n$ can be represented by a univariate polynomial through an isomorphism $\mathbb {F}_2^n \cong \mathbb {F}_{2^n}$. This representation can be used to attack cryptographic ciphers (see, e.g., [6] and [15]). We study these univariate representations for $\chi _n$ to give insight in these representations.

The formula for $\chi _n^{-1}$ [20] gives rise to a simple question, that we answer in this paper. How many monomials of a certain degree occur in this formula?

Lastly, we might consider using the rule $y_i = x_i + (x_{i+1}+1)x_{i+2}$ on field extensions (of $\mathbb {F}_2$) or finite fields of other characteristic.

*Our contributions*

We have studied the aforementioned algebraic properties and present the following results.

In Sect. 4, we discuss univariate polynomial expressions for the maps $\chi _n$. In particular, we show that for $n\ne 1,3$, they are not power functions. After that, we compute the number of different representations as a univariate polynomial with coefficients in the base field $\chi _n$ can take. This number is equal to $\underline{n}\cdot \varphi (n)$, where $\underline{n}$ is the number of normal elements in $\mathbb {F}_{2^n}$ and $\varphi (n) = \#(\mathbb {Z}/n\mathbb {Z}^*)$. Lastly, we give bounds on the degree and sparsity of $\chi _n$ when given as a univariate polynomial.

Secondly, based on [20], we considered that there was no formula known for the number of monomials of a given degree in $\chi _n^{-1}$. We compute those in Sect. 5. They behave according to binomial coefficients, i.e., the number of monomials of degree $m>0$ in $\chi _n^{-1}$ is equal to $$\left( {\begin{array}{c}\frac{n+1}{2}\\ m\end{array}}\right) $$.

Thirdly, in Sect. 6, we view $\chi _n$ as a polynomial map (see [31]), and from that conclude that, if we take the same rule to define a $\chi _n^{(d)}$ over $\mathbb {F}_{2^d}$, it cannot be invertible for some *d*. We show that for even *d* and all *d* with $d\equiv 0 \pmod {3}$, the map $\chi _n^{(d)}$ is not invertible, and conjecture that this holds for any $d > 1$.

We finalize this section by showing that the same rule will not give an invertible map in characteristic $p>2$.

## Notations and conventions

We write $\mathbb {F}_2$ for the finite field of two elements and $\mathbb {F}_m$ for a (finite) field of *m* elements. Additionally, we have the notation $\mathbb {F}_2^n$ for the standard *n*-dimensional $\mathbb {F}_2$-vector space, obtained as the Cartesian product of *n* copies of $\mathbb {F}_2$.

We write $0^n$ for the zero vector of *n* zeroes, and $1^n$ for the all-one vector of *n* ones. In general if we write any string of bits *s* in the form $s^n$, we mean the concatenation of that string to itself *n* times.

The number of 1s in a sequence or vector *x* is called the *Hamming weight* and is denoted as $\textrm{wt} (x)$.

We write  for the (sub-)space spanned by the vectors $v_1,\ldots ,v_n$.

We consider a basis to be an *ordered* set that is linearly independent and spanning. Therefore, we write them as tuples.

Thus  give rise to isomorphic vector spaces, although we do consider the bases $(v_1,\ldots ,v_n)$ and $(v_2,v_1,v_3,\ldots ,v_n)$ distinct.

We write $\textrm{lg}$ for the binary logarithm and $R^*$ for the group of units of the ring *R*.

For a polynomial ring in one indeterminate *X* with coefficients in *R*, we write *R*[*X*] and likewise for a polynomial ring over *n* indeterminates $X_1,\ldots ,X_n$, we write $R[X_1,\ldots ,X_n]$.

For any positive integer *n*, we denote the number of elements in $\mathbb {Z}/n\mathbb {Z}^*$ by $\varphi (n)$, the Euler totient function.

## $\chi _n$ and preliminary results

In this paper we study the maps $\chi _n$:

### Definition 1

($\chi _n$) Let $n\ge 1$. The map $\chi _n :\mathbb {F}_2^n \rightarrow \mathbb {F}_2^n,\ x \mapsto y$ is given by $y_i = x_i + (x_{i+1}+1)x_{i+2} = x_i + x_{i+1}x_{i+2} + x_{i+2}$ where the indices are taken modulo *n*.

We see that each $\chi _n$ is a map of (algebraic) degree 2.

### Shift maps and shift-invariant maps

A class of maps that is of interest with respect to $\chi$ is the class of shift maps.

#### Definition 2

(*Shift maps*) For any $n\ge 1$ and any $k\ge 0$ we can define two maps  and  on $\mathbb {F}_2^n$, by iteratingWe have  and .

#### Definition 3

(*Shift-invariant maps*) A map $F:\mathbb {F}_2^n \rightarrow \mathbb {F}_2^n$ is called *shift invariant* if we have  for all $k \ge 0$.

By induction, we can relax the criterium for shift-invariance:

#### Lemma 1

Similarly, a map $F:\mathbb {F}_2^n \rightarrow \mathbb {F}_2^n$ is shift invariant if we have .

Using that , one can find the following generalization of Lemma 1.

#### Lemma 2

Let $F:\mathbb {F}_2^n \rightarrow \mathbb {F}_2^n$ be a map, let $k\ge 1$ be such that $\gcd (k,n)=1$ and . Then *F* is shift invariant.

#### Proof

Since $\gcd (k,n)=1$, there exist integers *a*, *l* such that $ak = 1 + ln$. By induction to *a*, we know that . Hence . Since , we find that  and we are done by Lemma 1. $\square$

#### Lemma 3

For each *n*, $\chi _n :\mathbb {F}_2^n \rightarrow \mathbb {F}_2^n$ is shift invariant.

**Fig. 1:**
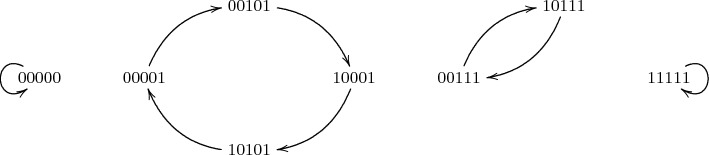
Transformation of some binary vectors under $\chi _5$

As an example, we give a graph of $\chi _5$ in Fig. 1. Since $\chi _5$ is shift invariant, for every input, the output can be deduced from this graph.

### Invertibility and order

From [8], we know that $\chi _n$ is invertible if and only if *n* is odd. Furthermore, we have a formula for the order of $\chi _n$, as a bijection in the group of bijections on $\mathbb {F}_2^n$, in this case.

#### Theorem 1

(Order of $\chi _n$ ([30])) Let $n>0$ be an odd integer. Then $\textrm{ord} (\chi _n) = 2^{\lceil \textrm{lg} (\frac{n+1}{2})\rceil }$.

In particular, we find that repeating $\chi _n$ for $2^{\lceil \textrm{lg} (\frac{n+1}{2})\rceil }-1$ times, then this gives a way for computing the inverse. A direct formula for the inverse is determined in [20].

## Univariate representations of $\chi _n$

**Fig. 2:**
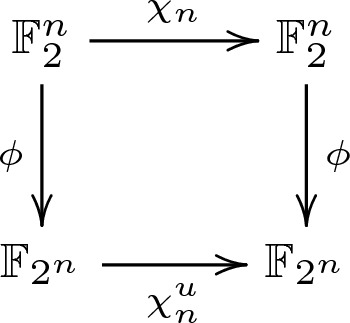
The schematics for the univariate $\chi _n$

We can choose any isomorphism $\mathbb {F}_2^n \overset{\phi }{\cong }\ \mathbb {F}_{2^n}$ and consider $\chi ^u_n :\mathbb {F}_{2^n} \rightarrow \mathbb {F}_{2^n}$ that is given by $\chi ^u_n:= \phi \circ \chi _n \circ \phi ^{-1}$, as depicted in Fig. 2.

This $\chi ^u_n$ can be written as a univariate polynomial with coefficients in $\mathbb {F}_{2^n}$ by using Lagrange interpolation on all inputs. (See [32] and [19] (Thm 1.71).) With Lagrange interpolation on all pairs $(x_i,\chi _n(x_i))$ one will find a polynomial $f(X)\in \mathbb {F}_{2^n}[X]$ that satisfies $f(x_i) = \chi _n(x_i)$ for all $x_i$ and has degree $<q^n$. Note that by performing the interpolation on all inputs, one does not have to compute inverses, as:

$$\begin{aligned} f(t) = \sum _{i=0}^{2^n-1} f(x_i)\cdot \ell _i(t), \qquad \qquad \ell _i(t) = \prod _{\begin{array}{c} i=0,\ldots ,2^n-1 \\ i\ne j \end{array}} \frac{t-x_i}{x_j-x_i} \end{aligned}$$

and we have

$$\begin{aligned} \prod _{\begin{array}{c} i=0,\ldots ,2^n-1 \\ i\ne j \end{array}} x_j - x_i = \prod _{\beta \in \mathbb {F}_{2^n}^*} \beta = \gamma ^{\sum _{i=0}^{2^n-2} i} = \gamma ^{\frac{1}{2}(2^n-2)(2^n-1)} = 1, \end{aligned}$$

where $\gamma$ is some generator of $\mathbb {F}_{2^n}^*$.

A polynomial $f(X) \in \mathbb {F}_{q^n}[X]$ is a *permutation polynomial* if its corresponding polynomial functions $t \mapsto f(t)$ is a permutation of $\mathbb {F}_{q^n}$. Two polynomials $f(X),g(X) \in \mathbb {F}_{q^n}[X]$ are *functionally equivalent* if their corresponding polynomial functions $t\mapsto f(t)$ and $t \mapsto g(t)$ satisfy $f(t) = g(t)$ for all $t\in \mathbb {F}_{q^n}$. It is straightforward that this is an equivalence relation. Equivalently, two polynomials $f(X), g(X) \in \mathbb {F}_{q^n}[X]$ are functionally equivalent if and only if $f(X) \equiv g(X) \pmod {X^{q^n}-X}$. (See [19] 7.2) Thus, there always is a representative of degree $<q^n$.

We now give an example where we use Lagrange interpolation to find a polynomial representation of $\chi _3$:

### Example 1

Consider $\chi _3 :\mathbb {F}_2^3 \rightarrow \mathbb {F}_2^3$ and the finite field $\mathbb {F}_{2^3}:= \mathbb {F}_2(\alpha ) = \mathbb {F}_2[X]/(X^3+X+1)$. Let $(1,\alpha ,\alpha ^2)$ be an ordered basis, then an isomorphism of vector spaces can be found as

$$\begin{aligned} \phi :\mathbb {F}_2^3 \rightarrow \mathbb {F}_{2^3},\ (x_0,x_1,x_2) \mapsto x_0 + \alpha \cdot x_1 + \alpha ^2\cdot x_2. \end{aligned}$$

Then $\chi ^u_3:= \phi \circ \chi _3\circ \phi ^{-1}$ is given by: $0\mapsto 0$, $1\mapsto \alpha ^3$, $\alpha \mapsto \alpha ^4$, $\alpha ^2 \mapsto \alpha ^6$, $\alpha ^3 \mapsto 1$, $\alpha ^4 \mapsto \alpha$, $\alpha ^5 \mapsto \alpha ^5$ and $\alpha ^6 \mapsto \alpha ^2$. By using Lagrange interpolation, we find $\chi ^u_3(X) \in \mathbb {F}_{2^3}[X]$ as

$$\begin{aligned} \chi ^u_3(X) = \alpha ^3 X^6 + \alpha ^5 X^5 + \alpha ^2 X^4 + \alpha ^6 X^3 + \alpha X^2 + \alpha ^2 X. \end{aligned}$$

### Power functions

A special kind of polynomials are those whose representative consists of a single monomial.

#### Definition 4

(*Power functions*) A *power function* is a polynomial function that can be represented by a single monomial in $\mathbb {F}_{q^n}[X]$. We write $(\cdot )^{e} :\mathbb {F}_{q^n} \rightarrow \mathbb {F}_{q^n}$ for a power function, here $e\ge 0$.

Since $\mathbb {F}_{q^n}^*$ is cyclic of order $q^n-1$, we find that $t^{q^n-1} = 1$ for all $t\in \mathbb {F}_{q^n}^*$, hence $t^{q^n} = t$ for all $t\in \mathbb {F}_{q^n}$. Therefore, we only need to consider power functions with $0 \le e < q^n-1$. A power function is not necessarily a permutation polynomial.

#### Proposition 1

(Bijectivity ([19] 7.8)) A power function $(\cdot )^{e} :\mathbb {F}_{q^n} \rightarrow \mathbb {F}_{q^n}$ is a permutation polynomial if and only if $\gcd (e,q^n-1) = 1$.

The set of all bijective power functions forms a group of order $\varphi (q^n-1)$, which we denote as $\textrm{Pow}(\mathbb {F}_{2^n})$. It is isomorphic to the automorphism group of $\mathbb {F}_{q^n}^*$, denoted as $\textrm{Aut}(\mathbb {F}_{q^n}^*)$ (see [1] or [19] Ex 2.20).

It is also easy to express the order of a power function, as in the group of bijective power functions.

#### Proposition 2

(Order of power function) The order of the power function $(\cdot )^{e}$ on $\mathbb {F}_{q^n}$ is given by the (multiplicative) order of *e* in $\mathbb {Z}/(q^n-1)\mathbb {Z}$.

#### Proof

Note that $(\cdot )^{e} \circ (\cdot )^{e} = (\cdot )^{e^2}$, and similarly for *k* compositions: $(\cdot )^{e^k}$. $\square$

### Normal bases

#### Definition 5

(*Normal basis* [26]) Consider $\mathbb {F}_q \subset \mathbb {F}_{q^n}$. Then $\beta \in \mathbb {F}_{q^n}$ is called a *normal element* of $\mathbb {F}_{q^n}$ over $\mathbb {F}_q$ if the set $\{\beta , \beta ^q, \beta ^{q^2}, \ldots , \beta ^{q^{n-1}} \}$ is a linearly independent set. When considered as a tuple, this tuple is called a *normal basis* of $\mathbb {F}_{q^n}$ over $\mathbb {F}_q$.

Each element in a normal basis is a normal element. In [17] it is first proven that every finite extension field has a normal basis. In [26] the result is extended to giving the number of normal elements. In the following, when we will omit the *over *$\mathbb {F}_q$ and write $\beta$ is a normal element of $\mathbb {F}_{q^n}$, or *S* is a normal basis of $\mathbb {F}_{q^n}$, when it is clear that they are considered *over *$\mathbb {F}_q$.

#### Example 2

Consider $\mathbb {F}_2\subset \mathbb {F}_8$, with $\mathbb {F}_8:= \mathbb {F}_2(\alpha ) = \mathbb {F}_2[X]/(X^3+X+1)$. Then $\alpha ^3$ is a normal element of $\mathbb {F}_8$:Therefore the tuple $(\alpha ^3, \alpha ^6, \alpha ^5)$ is a normal basis. These normal elements are roots of $X^3+X^2+1$.

With any choice of a normal element (and its corresponding normal basis) one obtains an isomorphism between $\mathbb {F}_q^n$ and $\mathbb {F}_{q^n}$, as follows:

1 $$\begin{aligned} \phi _{\beta } :\mathbb {F}_q^n \rightarrow \mathbb {F}_{q^n},\ (x_0,\ldots ,x_{n-1}) \mapsto x_0 \beta + \ldots + x_{n-1} \beta ^{q^{n-1}}. \end{aligned}$$

With the isomorphism $\phi _{\beta }$, taking the *q*th power in $\mathbb {F}_{q^n}$ of an element corresponds to a shift of the coordinates in $\mathbb {F}_q^n$ in the following way:

#### Lemma 4

([28] Lemma 5) Let $\beta$ be a normal element of $\mathbb {F}_{q^n}$. Let $\phi _{\beta }$ be as in (1). Then .

We now give an example of the representation of $\chi _3$ as a univariate polynomial.

#### Example 3

Consider the map $\chi _3$. Let $\alpha ^3$ be a normal element in $\mathbb {F}_{2^3}$ as in Example 2. We define $\chi ^u_3:= \phi _{\alpha ^3} \circ \chi _3 \circ \phi _{\alpha ^3}^{-1}$ with its inputs and outputs as given in columns 3 and 4 of Table 1. By using Lagrange interpolation we find that $\chi ^u_3(t) = t^6$ for all *t*.

**Table 1:** The maps $\chi _3$ and $\chi ^u_3$

$(a_0,a_1,a_2)$	$\chi _3(a_0,a_1,a_2)$	$\phi _{\alpha _3}(a_0,a_1,a_2)$	$\phi _{\alpha _3}(\chi _3(a_0,a_1,a_2))$
(0, 0, 0)	(0, 0, 0)	0	0
(0, 0, 1)	(1, 0, 1)	$\alpha ^5$	$\alpha ^2$
(0, 1, 0)	(0, 1, 1)	$\alpha ^6$	$\alpha$
(0, 1, 1)	(0, 1, 0)	$\alpha$	$\alpha ^6$
(1, 0, 0)	(1, 1, 0)	$\alpha ^3$	$\alpha ^4$
(1, 0, 1)	(0, 0, 1)	$\alpha ^2$	$\alpha ^5$
(1, 1, 0)	(1, 0, 0)	$\alpha ^4$	$\alpha ^3$
(1, 1, 1)	(1, 1, 1)	1	1

We saw that $\chi ^u_3(X)\in \mathbb {F}_2[X]$ in the previous example. We prove the more general theorem that any shift-invariant map has a univariate representation with coefficients in the base field.

#### Theorem 2

Let $F:\mathbb {F}_q^n \rightarrow \mathbb {F}_q^n$ be a shift-invariant map. Let $\beta$ be a normal element of $\mathbb {F}_{q^n}$ and $\phi _{\beta }$ as in (1). Consider the map $F^u:\mathbb {F}_{q^n} \rightarrow \mathbb {F}_{q^n}$ defined by $F^u:= \phi _{\beta } \circ F \circ \phi _{\beta }^{-1}$. Then $F^u$ is a polynomial function with $F^u(X) \in \mathbb {F}_q[X]$.

#### Proof

By Lemma 4 we find that $F^u(X^q) = F^u(X)^q$ since *F* is shift invariant. If we then write $F^u \in \mathbb {F}_{q^n}[X]$ as $\sum _{i=0}^m a_iX^i$ for some *m*, then we have

$$\begin{aligned} \sum _{i=0}^m a_i X^{iq} = F^u(X^q) = F^u(X)^q = \sum _{i=0}^m a_i^q X^{iq}. \end{aligned}$$

Hence, $a_i^q = a_i$ for all $i=0,\ldots ,m$ and thus $F^u(X)\in \mathbb {F}_q[X]$. $\square$

Since $\chi _n$ is a shift-invariant map, we have the following immediate corollary:

#### Corollary 1

$$\chi ^u_n(X) \in \mathbb {F}_2[X]$$.

### The map $\chi ^u_n$ is only a power function for $n=1,3$

The map $\chi _1$ is the identity function, hence is equivalent to the power function with $e=1$. We also found that for a suitable choice of normal basis, $\chi ^u_3(X) = X^6$, a power function.

It is easy to see that for even *n* there is no power function equivalent to $\chi ^u_n(X)$.

#### Lemma 5

For any even *n*, there is no normal basis representation such that $\chi ^u_n$ is a power function.

#### Proof

Suppose that there exists a normal basis representation such that $\chi ^u_n$ is a power function. Since $\chi _n((01)^{n/2}) = 0^n$, there needs to exist some nonzero $\alpha \in \mathbb {F}_{2^n}$ with $\alpha ^s = 0$ for some integer *s*, a contradiction. $\square$

If $n>3$ is a Mersenne-exponent, i.e., $2^n-1$ is a prime number, then it is also easy to show that $\chi ^u_n$ is not a power function.

#### Proposition 3

(Excluding Mersenne-exponents) If $n>3$ is such that $2^n-1$ is a prime number, then there exists no normal basis representation of $\chi _n$ such that $\chi ^u_n$ is a power function.

#### Proof

Since the order of a group element is preserved under isomorphism, we inspect the order of $\chi _n$ and power functions. Since $2^n-1$ is a prime number, then $\varphi (2^n-1) = 2^n-2$. Therefore, the only possibilities for the order of a power function are divisors of $2^n-2$. By Theorem 1, the order of $\chi _n$ is divisible by 4 for all $n>3$. The expression $2^n-2$ has at most one factor 2, so there exists no power function that is equivalent to $\chi _n$. $\square$

For $n = 3$, we have $2^3 - 1 = 7$, a prime number. However, $\varphi (7) = 2\cdot 3$ and $\chi _3$ has order 2, so the proof of Proposition 3 does not hold for $\chi _3$.

For the general case, we can prove that $\chi ^u_n$ is not a power function by computing differential probabilities.

#### Differential probabilities

In this paragraph, we discuss differential probabilities, and with that show that $\chi _n$ is only a power function for $n=1,3$. Differential probabilities were studied in [3] as a way of breaking the cipher DES [24].

##### Definition 6

(*Differential probability*) Let $f:G \rightarrow H$ be a map between finite (additive) groups *G* and *H*. Let $g\in G$ and $h\in H$ be arbitrary. Then we define the *differential probability of* *f* *at * (*g*, *h*) as

$$\begin{aligned} \textrm{DP}_f(g,h) = \#\{ x\in G \mid f(x) - f(x - g) = h \}/|G|. \end{aligned}$$

Since we have mostly characteristic 2 in this section, the −-signs can be replaced by $+$-signs.

##### Example 4

(Differential distribution table of $\chi _3$) Consider $\chi _3 :\mathbb {F}_2^3 \rightarrow \mathbb {F}_2^3$, then we compute $\textrm{DP}_{\chi _3}(g,h)$ for all $g,h \in \mathbb {F}_2^3$ and put them in a table, where the rows are indexed by *g* and columns are indexed by *h*. The dashes represent 0. Each entry in the table, $\textrm{DDT}_{gh}$, represents $\#\mathbb {F}_2^3\cdot \textrm{DP}_{\chi _3}(g,h)$ (see Table 2). Such a table we call a *differential distribution table*.

**Table 2:** Differential distribution table (DDT) of $\chi _3$

	Output difference								
	$\chi _3$	000	001	010	011	100	101	110	111
Input difference	000	8	–	–	–	–	–	–	–
	001	–	2	–	2	–	2	–	2
	010	–	–	2	2	–	–	2	2
	011	–	2	2	–	–	2	2	–
	100	–	–	–	–	2	2	2	2
	101	–	2	–	2	2	–	2	–
	110	–	–	2	2	2	2	–	–
	111	–	2	2	–	2	–	–	2

In the next proposition we will show that the DDT is an invariant for (Boolean) functions.

##### Proposition 4

[Differential probabilities under linear isomorphisms] Let $G \overset{\phi }{\cong }\ H$ be isomorphic groups. Let $f:G \rightarrow G$ be a map and let $\widehat{f}:H \rightarrow H$ be the map induced through the isomorphism. Then $\textrm{DP}_{\widehat{f}}(g,h) = \textrm{DP}_{f}(\phi ^{-1}(g),\phi ^{-1}(h))$ for all $g,h\in H$.

##### Proof

We have

$$\begin{aligned} \textrm{DP}_{\widehat{f}}(g,h)&= \#\{ x \in H \mid (\phi \circ f \circ \phi ^{-1})(x) - (\phi \circ f \circ \phi ^{-1})(x-g) = h \}/|H| \\&= \#\{ x\in H \mid (f\circ \phi ^{-1})(x) - f(\phi ^{-1}(x)- \phi ^{-1}(g)) = \phi ^{-1}(h) \}/|H| \\&= \#\{ y\in G \mid f(y) - f(y-\phi ^{-1}(g)) = \phi ^{-1}(h)\}/|G| \\&= \textrm{DP}_f(\phi ^{-1}(g),\phi ^{-1}(h)) \end{aligned}$$

for all $$g,h\in H$$. $$\square $$

One can similarly prove the following equalities for differential probabilities:

1. $$\textrm{DP}_{f+L}(g,h) = \textrm{DP}_f(g,h-L(g))$$;
2. $$\textrm{DP}_{f\circ L}(g,h) = \textrm{DP}_f(L(g),h)$$;
3. $$\textrm{DP}_{A\circ f}(g,h) = \textrm{DP}_f(g,A^{-1}(h))$$,

where the *L* and *A* are affine maps and *A* is, moreover, an invertible affine map. The differential properties of $\chi _n$ have been studied extensively (see [8, 9]). We say *h* is *compatible* with a *g* if $\textrm{DP}_{\chi _n}(g,h) \ne 0$.

In the following, we will write $a'$ and $b'$ instead of *g*, *h* to coincide with the standard notation, where $a'$ denotes an input difference, i.e., $a' = a + a^*$, and $b' = b + b^*$ an output difference. We will use the following result:

##### Proposition 5

(Differential probabilities for $\chi$ [8]) Let $n>1$ be an arbitrary odd integer and $a'\in \mathbb {F}_2^n$. Then for any $b'\in \mathbb {F}_2^n$ compatible with $a'$, we have $\textrm{DP}_{\chi _n}(a',b') = 2^{-w(a')}$, where

$$\begin{aligned} w(a') = {\left\{ \begin{array}{ll} n-1 &{} \hbox {if } a' = 1^n; \\ \textrm{wt} (a') + r_{a'} &{} \hbox {else.} \end{array}\right. } \end{aligned}$$

where $r_{a'}$ is the number of 001-subsequences in $a'$.

Since we have been unable to find a complete proof of this result in the literature,^1^ we include our own proof in Appendix 1.

For power functions, the differential probabilities have also been studied, in e.g., [4]:

##### Proposition 6

(Differential probabilities for power functions [4]) Let $0 \le e \le 2^n-1$ and let $f = (\cdot )^{e} :\mathbb {F}_{2^n} \rightarrow \mathbb {F}_{2^n}$ be a power function. Then $\textrm{DP}_f(a',b') = \textrm{DP}_f(ya',y^eb')$ for all $y\in \mathbb {F}_{2^n}^*$.

In particular, if we compute $\textrm{DP}_f(1,b')$ for all $b'$, we can use the above proposition to deduce the remainder of the differential distribution table. As a direct corollary, we see that the number of occurrences of 0 is the same in every row (except the first), and the same holds for the number of occurrences of $2,4,\ldots$.

##### Example 5

(Differential distribution table of $t\mapsto t^6$) Let $\mathbb {F}_8$ be determined by $X^3+X+1$ and consider $(\cdot )^{6} :\mathbb {F}_8 \rightarrow \mathbb {F}_8$. Then in Table 3, one sees the differential distribution table for $(\cdot )^{6}$.

**Table 3:** The DDT of $t\mapsto t^6$

	Output difference								
	$(\cdot )^{6}$	0	1	*x*	$x^2$	$x^3$	$x^4$	$x^5$	$x^6$
Input difference	0	8	–	–	–	–	–	–	–
	1	–	2	–	–	2	–	2	2
	*x*	–	–	–	2	–	2	2	2
	$x^2$	–	–	2	–	2	2	2	–
	$x^3$	–	2	–	2	2	2	–	–
	$x^4$	–	–	2	2	2	–	–	2
	$x^5$	–	2	2	2	–	–	2	–
	$x^6$	–	2	2	–	–	2	–	2

We can now use what we know about differential properties of $\chi _n$ and power functions to prove:

##### Theorem 3

[$\chi _n$ is not a power function for $n\ne 1,3$] Let $n\ne 1,3$ be a positive integer. Then there exists no way to write $\chi ^u_n$ as a power function.

##### Proof

Let $n\ne 1,3$ be an arbitrary odd positive integer. (The even case has been proven in Lemma 5.) Consider any isomorphism from $\mathbb {F}_2^n$ to $\mathbb {F}_{2^n}$ under which $\chi _n$ would become $\chi ^u_n$. By Proposition 4, we find that their differential distribution should be similar. Set $a' = 110^{n-2}$ and $a'' = 10^{n-1}$. Then we find that $\textrm{DP}_{\chi _n}(a',b') = \frac{1}{8}$ and $\textrm{DP}_{\chi _n}(a'',b') = \frac{1}{4}$ for all $b'$ that are compatible with $a',a''$ respectively, by Proposition 5. Whereas, by Proposition 6, we have that each row of the DDT should have the same number of occurrences of $0,2,4,\ldots$. Therefore, $\chi ^u_n$ cannot be a power function. $\square$

##### Definition 7

(*Extended affine equivalence*) Let *F* and *G* be two Boolean functions from $\mathbb {F}_2^n$ to $\mathbb {F}_2^m$. We say that *F* and *G* are *extended affine equivalent* if there exist:

- an affine permutation *A* of $\mathbb {F}_2^n$;
- an affine permutation *B* of $\mathbb {F}_2^m$; and
- an affine map $C :\mathbb {F}_2^n \rightarrow \mathbb {F}_2^m$,

such that $$G = (B\circ F \circ A) + C$$.

We obtain, by using the properties for differential probability listed after Proposition 4, as a direct corollary to Theorem 3:

##### Corollary 2

Let $n\ne 1,3$ be a positive integer. Let *F* be any extended affine equivalent of $\chi _n$. Then $F^u$ is not a power function.

### Number of different univariate polynomial representations of $\chi _n$.

A priori, since we make several choices, there could be many different univariate representations of $\chi _n$ for each *n*. In this section, we go over the choices we make and discuss how they affect the outcome of the univariate representation. In order, we discuss the choice of representation of the field, i.e., the irreducible polynomial of degree *n* that defines $\mathbb {F}_{2^n}$. After that, we treat how different normal elements may give rise to different univariate polynomial representations. Each normal element $\beta$ has a canonical ordered basis, yielding an isomorphism $\phi _{\beta }$ as in Eq. 1. But there might be basis transformations, that shuffle the basis elements. This will provide a different isomorphism from $\mathbb {F}_2^n$ to $\mathbb {F}_{2^n}$, and in some cases it will give a univariate polynomial in the base field.

*Choosing an irreducible polynomial to create the field extension*

It is a well-known result that for any prime power there exists (up to isomorphism) a unique field with that many elements. Does this “up to isomorphism” interfere with the univariate expression of a map? The isomorphism $\phi _{\beta }$ is defined by the normal element. This normal element is defined by being a root of a polynomial. In fact, if the degree of this polynomial is *d*, then there are *d* roots, all of which are normal elements.

#### Proposition 7

Let $\mathbb {F}_f:= \mathbb {F}_2[X]/(f(X))$ and $\mathbb {F}_g:= \mathbb {F}_2[X]/(g(X))$ be isomorphic fields. Let $\alpha$ be a normal element in $\mathbb {F}_f$ that is a root of the polynomial $h(X)\in \mathbb {F}_2[X]$. Then there exists some $\beta \in \mathbb {F}_g$ that is a normal element as a root of *h*(*X*). Furthermore, $\beta ,\beta ^2,\ldots ,\beta ^{2^{\deg f-1}}$ are all roots of *h*(*X*).

#### Proof

Let $\psi :\mathbb {F}_f \rightarrow \mathbb {F}_g$ be an isomorphism. Then since $h(\alpha ) = 0$, we must have $\psi (h(\alpha )) = \psi (0) = 0$. Since $\psi$ is a field-homomorphism, we find that $\psi (h(\alpha )) = h(\psi (\alpha ))$ as a polynomial equation consists solely of additions and multiplications. Therefore $\beta = \psi (\alpha )$ is also a root of *h*(*X*).

For the second statement we note that $(a+b)^{2^i} = a^{2^i}+b^{2^i}$ for $i\ge 0$ since we work in a field of characteristic 2. Therefore $h(\alpha ^{2^i}) = h(\alpha )^{2^i} = 0$ for all $i \in \{0,\ldots , \deg f-1\}$. $\square$

Since $\mathbb {F}_{2^n}^*$ is cyclic for any *n*, we find that any normal element generates the entire group. As the isomorphism $\psi$ maps normal elements to linear combinations between powers of the same normal element, we therefore find that the “up to isomorphism” indeed does not influence the univariate expression of a map.

*Choice of the normal element*

We have a choice on the normal elements that we make in defining a univariate expression. This choice of normal element influences the resulting univariate expression, in particular, if $\beta ,\gamma$ are two distinct normal elements such that $\gamma$ is not in any normal basis containing $\beta$, then we get different univariate polynomials.

From [19] (Thm 3.73), or [26], we obtain the following formula for the number of distinct normal elements:

#### Theorem 4

(Number of normal elements) Let *q* be a prime power and $m\ge 1$ an integer. There exist precisely $\Phi _q(X^m-1)/m$ normal elements in $\mathbb {F}_{q^m}$ (w.r.t. $\mathbb {F}_q$).

Here, $\Phi _q(f)$ denotes the number of polynomials in $\mathbb {F}_q[X]$ that are coprime to *f* and have a smaller degree than $\deg (f)$.

We will denote the number of normal elements in $\mathbb {F}_{2^n}$ (w.r.t. $\mathbb {F}_2$) by $\underline{n}$. Thus, $\underline{n} = \Phi _2(X^n-1)/n$.

*(Re-)Ordering the normal basis*

Given a normal basis $(\beta ,\beta ^q,\ldots ,\beta ^{q^{n-1}})$ of $\mathbb {F}_{q^n}$, there are several ways to re-order the elements in this basis. In particular, for every permutation $\sigma \in S_n:= \textrm{Sym}(\{0,\ldots ,n-1\})$ we have a re-ordered basis by $(\beta ^{\sigma (0)}, \beta ^{\sigma (1)},\ldots ,\beta ^{q^{\sigma (n-1)}})$.

Then we can define the isomorphism

2 $$\begin{aligned} \phi _{\beta }^{\sigma } :(x_0,\ldots ,x_{n-1}) \mapsto \sum _{i=0}^{n-1} x_i \beta ^{q^{\sigma (i)}} \end{aligned}$$

as the isomorphism corresponding to the one in (1) when the basis is re-ordered. (Note that the isomorphism given in (1) is the one where $\sigma$ is the identity permutation.) A priori therefore, there are *n*! different univariate representations when the normal basis is fixed.

We indicate that a left-shift over the basis elements corresponds with the permutation $$\sigma = \begin{pmatrix} 0&1&2&\cdots&n-1 \end{pmatrix}$$. We can therefore write . In the case that a map *F* is shift invariant, we can immediately reduce the number of representations to $(n-1)!$:

#### Lemma 6

Let $\beta$ be a normal element in $\mathbb {F}_{q^n}$ and $F:\mathbb {F}_q^n \rightarrow \mathbb {F}_q^n$ a shift-invariant map. Let $\phi := \phi _{\beta }$ be as in (1) and $k\in \{1,\ldots ,n-1\}$ be arbitrary. Consider the isomorphism . Write $F^u_{\psi }$ for the corresponding univariate representation of *F*. Then $F^u_{\psi } = F^u_{\phi }$.

#### Proof

Using the Lagrange interpolation formula, we getas required. $\square$

#### Remark 1

Since , we find that the univariate expression is invariant under a shift of the coefficients, as expected. Thus we can assume, without loss of generality, that $\sigma (0)=0$. The same result holds when we have a re-ordered normal basis, thus for $\phi _{\beta }^{\sigma }$.

We will now investigate which re-orderings yield univariate expressions with coefficients in the base field. In the proof of Theorem 2 we use Lemma 4. Therefore it is prudent to look for ismorphisms under which taking a *q*th power corresponds to some shift coprime in length to the dimension of the domain of*F*. (See Lemma 2.)

Let $\gcd (k,n)=1$. We want to solve the equation  for $\sigma \in S_n$. We first illustrate this with an example.

#### Example 6

Let *q* be an arbitrary prime power, $n=5$ and $k=3$. We have the following commuting diagram by hypothesis: 
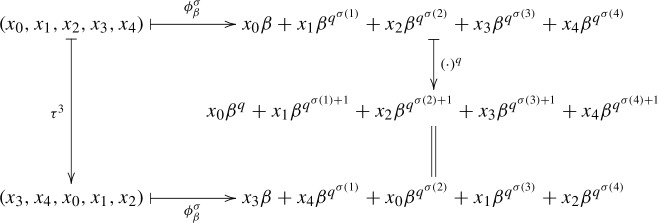
 From this diagram we find the following equations

$$\begin{aligned} 0 = \sigma (3)+1, \quad \sigma (1) = \sigma (4)+1, \quad \sigma (2) = 1, \quad \sigma (3) = \sigma (1)+1, \quad \sigma (4) = \sigma (2)+1. \end{aligned}$$

Therefore, we easily obtain $\sigma = (1\ 3 \ 4 \ 2)$.

#### Lemma 7

Consider a finite field extension $\mathbb {F}_{q^n}$ of $\mathbb {F}_q$ with a normal element $\beta$. Let $0 \le k \le n-1$ be such that $\gcd (k,n)=1$. Then there exists a unique $\sigma$ such that .

#### Proof

Write $\textbf{x}$ for the vector $(x_0,\ldots ,x_{n-1})$. We have $\phi _{\beta }^{\sigma }(\textbf{x}) = \sum _{i=0}^{n-1} x_i \beta ^{q^{\sigma (i)}}$ andThen from the hypothesis $\gcd (k,n)$, we find that  we find that, for indices $j, j+k$ modulo *n*, $\sigma (j+k) = \sigma (j)+1$. Since by Lemma 6 we can take $\sigma (0)=0$, we can deduce $\sigma (k) = 1$ and $\sigma (n-k) = n-1$. Since *k* is invertible in $\mathbb {Z}/n\mathbb {Z}$, the entire structure of $\sigma$ is then uniquely determined. $\square$

We conclude that given an irreducible polynomial and a normal element, there are $\varphi (n) = \#(\mathbb {Z}/n\mathbb {Z}^*)$ different univariate polynomial representations with coefficients in the prime field.

Taking into account the number of different normal elements, we obtain:

#### Theorem 5

Let $n>0$ be an arbitrary odd integer. Then there are $\underline{n}\cdot \varphi (n)$ different univariate polynomial representations of $\chi _n$ with coefficients in $\mathbb {F}_2$.

Some numbers of different univariate polynomial representations of $\chi _n$:

**Table 4:** The number of different univariate polynomial representations of $\chi _n$

n	3	5	7	9	11	13	15	17
$\underline{n}$	1	3	7	21	93	315	675	3825
$\varphi (n)$	2	4	6	6	10	12	8	16
$\underline{n}\cdot \varphi (n)$	2	12	42	126	930	3780	5400	61,200

### Bounds on degrees and sparsity

Irrespective of any choices, we can easily give bounds on the degree of the univariate expression and the sparsity of the univariate expressions.

We have various notions of degree. For instance, if we write $\chi _3$ as in Definition 1, we see that $\chi _3$ has degree 2. However, if we consider $\chi _3$ as a univariate polynomial as in Example 1, we see that $\chi _3(X)$ has degree 6. In order to make some sense of this, we explain the several different notions of degree.

Let $F :\mathbb {F}_2^n \rightarrow \mathbb {F}_2^m$ be a (Boolean) map in its *algebraic normal form*, that is, each $F_i$ is given as a multivariate polynomial in *n* indeterminates, that is a sum of monomials. Then, the degree of a coordinate function $F_i$ is the maximum of the degrees of its monomials. A monomial $X_1^{e_1}\cdots X_r^{e_r}$ has degree $e_1 + \ldots + e_r$. Then the *algebraic degree* of *F*, denoted by $\textrm{deg}_a (F)$, is the maximum of the degrees of each of the coordinate functions $F_i$.

When $m=1$, the algebraic degree corresponds with the usual degree.

A second notion of degree is applicable to a map $F :\mathbb {F}_{2^n} \rightarrow \mathbb {F}_{2^n}$ that is given by a univariate polynomial. Write $F(X) = \sum _{j=0}^{2^n-1} c_j X^j$. Then the 2-*degree* of *F* is given by

$$\begin{aligned} \textrm{deg}_2 (F) = \max \{ \textrm{w}_2 (j) \mid 0 \le j \le 2^n-1, c_j \ne 0\}, \end{aligned}$$

where $\textrm{w}_2 (j)$ is the Hamming-weight of *j* in its binary expansion. The usual degree of a polynomial is the same as above, for the degree of a coordinate function.

#### Example 7

We continue from Example 1. We see that the exponents of *X* where there is a non-zero coefficient are 6, 5, 4, 3, 1. The list of their respective $\textrm{w}_2 (j)$ is 2, 2, 1, 2, 1. Hence we see that the 2-degree of $\chi ^u_3$ is 2.

We see that in the example, the 2-degree of $\chi ^u_3$ is the same as the algebraic degree of $\chi _3$. This holds in general (see [5]).

The bounds that we are going to prove in this section are on the regular degree of the univariate polynomial. Since we know that $\chi _n$ has algebraic degree 2, we know that its 2-degree should be 2 as well. This means that the only powers of *t* in $\chi ^u_n(t)$ have Hamming-weight at most 2. The largest possible such number is then $2^{n-1}+2^{n-2}$, since the powers of *t* are already bounded by $2^n-1$. Likewise the lowest possible degree for $\chi ^u_n(t)$ is 3. We have

$$\begin{aligned} 3 \le \deg \chi ^u_n(t) \le 2^{n-1}+2^{n-2}. \end{aligned}$$

By the same line of reasoning, we have an immediate formula for the sparsity of $\chi ^u_n(t)$, by the 2-degree. We obtain that the number of monomials in $\chi ^u_n(t)$ is at most $$\left( {\begin{array}{c}n\\ 1\end{array}}\right) + \left( {\begin{array}{c}n\\ 2\end{array}}\right) $$. Each possible exponent can be written in a binary sequence of length *n*. We allow only those where there is one 1, or two 1s, as there is no constant term in the ANF of $\chi _n$.

In Appendix 2, we give a table of the minimum and maximum sparsity of (actually occurring) univariate expressions of $\chi _n$, as well as the minimum and maximum occurring degrees.

We furthermore list the univariate polynomial representations of $\chi _n$ for $n\le 7$.

## Monomial count of $\chi _n^{-1}$

We find in [20] that the inverse of $\chi _n^{-1}$ has a nice expression:

### Theorem 6

[$\chi _n^{-1}$ ([20])] For odd $n>0$, the formula for $\chi _n^{-1}$ is given by:

$$\begin{aligned} x_i = y_i + \sum _{j=1}^{(n-1)/2} y_{i-2j+1} \prod _{k=j}^{(n-1)/2} (y_{i-2k}+1), \end{aligned}$$

again, the indices are computed modulo *n*.

The degree of $\chi _n^{-1}$ is thus $(n+1)/2$.

For some use-cases, having this formula and its degree is enough as exhibited in [20]. However, for several cases, like algebraic attacks, one might use the monomial count, e.g., [12]. In any case, it is an interesting number to compute, and it turns out to follow a beautiful formula. We investigate in this section the total monomial count, and the number of monomials of a given degree in any one of the coordinates of $\chi _n^{-1}$.

In the following, we write ${\mathcal {M}}_e(f_i)$ for the set of monomials of degree *e* in the component $f_i$.

From Theorem 6, we can determine the following:

### Proposition 8

(Monomial count of $\chi _n^{-1}$) For each odd $n>0$ and each $0<m\le \frac{n+1}{2}$, we have

$$\begin{aligned} \# {\mathcal {M}}_m(\chi _{n,i}^{-1}) = \left( {\begin{array}{c}\frac{n+1}{2}\\ m\end{array}}\right) . \end{aligned}$$

For the proof, we use the following combinatorial lemma, which is a repeated application of Pascal’s Rule [27], and is very similar to the Hockey Stick Identity [18]:

### Lemma 8

Let *n* be a positive integer. Then for all $0\le k < n$ we have

3 $$\begin{aligned} \sum _{i=0}^k \left( {\begin{array}{c}n-i\\ k-i\end{array}}\right) = \left( {\begin{array}{c}n+1\\ k\end{array}}\right) . \end{aligned}$$

### Remark 2

Using the rule $$\left( {\begin{array}{c}n\\ k\end{array}}\right) = \left( {\begin{array}{c}n\\ n-k\end{array}}\right) $$ we also get the following formula:

$$\begin{aligned} \sum _{i=0}^{n-j} \left( {\begin{array}{c}n-i\\ j\end{array}}\right) = \left( {\begin{array}{c}n+1\\ j+1\end{array}}\right) . \end{aligned}$$

### Proof

(of Proposition 8) Let $h = \frac{n-1}{2}$. By working through the summation symbol, we find the numbers as in Table 5.

For instance, to count the number of monomials of degree $h-3$ that occur in the summation when $j=2$, we note that we have $h-1$ terms in the product, where at each time we have either the constant 1-term, or the degree-1-term $y_{i-2k}$. To get a degree of $h-3$, we need to have precisely two times the constant 1-term, or - in other words - $h-3$ times the degree-1-term $y_{i-2k}$ (varying indices). The number of possibilities is then given by $$\left( {\begin{array}{c}h-1\\ h-3\end{array}}\right) $$.

Or, to count the number of monomials of degree 3 that occur when $j=4$, we have in the product exactly $h-3$ terms. Of those, precisely two times we must choose, the degree-1-term, or, precisely $h-5$ times the constant term.

Finally, $\text {m}_i = \# {\mathcal {M}}(\chi _{n,i}^{-1})$ is the sum of all numbers in the column of $\text {m}_i$.

By Lemma 8, or, equivalently, the formula in the remark after this lemma, we then find the desired equalities, except for $\text {m}_1$, where we need to add the single degree-1-monomial $y_i$. $\square$

**Table 5:** The numbers $\text {m}_i$ of monomials of degree *i*, for each summand *j*. Here $h = \frac{n-1}{2}$

*j*	$\text {m}_{h+1}$	$\text {m}_{h}$	$\text {m}_{h-1}$	$\text {m}_{h-2}$	$\text {m}_{h-3}$	$\cdots$	$\text {m}_4$	$\text {m}_3$	$\text {m}_{2}$	$\text {m}_1$
$j=1$	1	$$\left( {\begin{array}{c}h\\ h-1\end{array}}\right) $$	$$\left( {\begin{array}{c}h\\ h-2\end{array}}\right) $$	$$\left( {\begin{array}{c}h\\ h-3\end{array}}\right) $$	$$\left( {\begin{array}{c}h\\ h-4\end{array}}\right) $$	$\cdots$	$$\left( {\begin{array}{c}h\\ 3\end{array}}\right) $$	$$\left( {\begin{array}{c}h\\ 2\end{array}}\right) $$	$$\left( {\begin{array}{c}h\\ 1\end{array}}\right) $$	1
$j=2$	–	1	$$\left( {\begin{array}{c}h-1\\ h-1\end{array}}\right) $$	$$\left( {\begin{array}{c}h-1\\ h-2\end{array}}\right) $$	$$\left( {\begin{array}{c}h-1\\ h-3\end{array}}\right) $$	$\cdots$	$$\left( {\begin{array}{c}h-1\\ 3\end{array}}\right) $$	$$\left( {\begin{array}{c}h-1\\ 2\end{array}}\right) $$	$$\left( {\begin{array}{c}h-1\\ 1\end{array}}\right) $$	1
$j=3$	–	–	1	$$\left( {\begin{array}{c}h-2\\ h-1\end{array}}\right) $$	$$\left( {\begin{array}{c}h-2\\ h-2\end{array}}\right) $$	$\cdots$	$$\left( {\begin{array}{c}h-2\\ 3\end{array}}\right) $$	$$\left( {\begin{array}{c}h-2\\ 2\end{array}}\right) $$	$$\left( {\begin{array}{c}h-2\\ 1\end{array}}\right) $$	1
$j=4$	–	–	–	1	$$\left( {\begin{array}{c}h-3\\ h-1\end{array}}\right) $$	$\cdots$	$$\left( {\begin{array}{c}h-3\\ 3\end{array}}\right) $$	$$\left( {\begin{array}{c}h-3\\ 2\end{array}}\right) $$	$$\left( {\begin{array}{c}h-3\\ 1\end{array}}\right) $$	1
$\vdots$	$\vdots$	$\vdots$	$\vdots$	$\vdots$	$\vdots$	$\vdots$	$\vdots$	$\vdots$	$\vdots$	$\vdots$
$j=h$	–	–	–	–	–	$\cdots$	–	–	1	1

Since we have determined the number of monomials of each degree $1 \le m < \frac{n+1}{2}$, we can immediately deduce the total number of monomials in any coordinate of $\chi _n^{-1}$.

### Corollary 3

(Monomials in $\chi _n^{-1}$) Let $n>0$ be odd, then the total number of monomials in any coordinate of $\chi _n^{-1}$ is equal to $2^{\frac{n+1}{2}}-1$.

## $\chi$ as a polynomial map

In this section, we investigate whether the function rule determined by $y_i \mapsto x_i + (x_{i+1}+1)x_{i+2}$, will yield invertible maps on other finite fields. We therefore take the most general form of a map that has this function rule; polynomial maps.

### Definition 8

(*Polynomial map*) Let $\mathbb {F}$ be an arbitrary field, and $\mathbb {F}[X_1,\ldots ,X_n]$ be the polynomial ring in *n* indeterminates. A *polynomial map* is a map $F = (F_1,\ldots ,F_n) :\mathbb {F}^n \rightarrow \mathbb {F}^n$ of the form

$$\begin{aligned} (x_1,\ldots ,x_n) \mapsto (F_1(x_1,\ldots ,x_n),\ldots ,F_n(x_1,\ldots ,x_n)), \end{aligned}$$

where each $$F_i \in \mathbb {F}[X_1,\ldots ,X_n]$$.

We can observe the related polynomial map of $\chi _n$ in *n* indeterminates. Here the field $\mathbb {F}$ that we look into is $\mathbb {F}_2$. This is given by

$$\begin{aligned} \Xi _n(X_1,\ldots ,X_n) = (X_1 + (X_2+1)X_3,X_2+(X_3+1)X_4,\ldots ,X_n + (X_1+1)X_2). \end{aligned}$$

A polynomial map is invertible if there exists a polynomial map $G:k^n \rightarrow k^n$ such that

$$\begin{aligned} X_i = G_i(F_1,\ldots ,F_n), \end{aligned}$$

for all $1\le i \le n$. By checking the determinant of the Jacobian of $\Xi _n$, we can check whether $\Xi _n$ is invertible.

For $\chi _n$ we have the following form for the Jacobian:

$$\begin{aligned} \textrm{Jac}_{\Xi _n} = \begin{pmatrix} 1 &{} X_3 &{} X_2+1 &{} 0 &{} 0 &{} \cdots &{} 0 \\ 0 &{} 1 &{} X_4 &{} X_3+1 &{} 0 &{} \cdots &{} 0 \\ 0 &{} 0 &{} 1 &{} X_5 &{} X_4+1 &{} \cdots &{} 0 \\ \vdots &{} \vdots &{} \vdots &{} \vdots &{} \vdots &{} &{} \vdots \\ X_n+1 &{} 0 &{} 0 &{} 0 &{} 0 &{} \cdots &{} X_1 \\ X_2 &{} X_1 + 1 &{} 0 &{} 0 &{} 0 &{} \cdots &{} 1 \end{pmatrix} \end{aligned}$$

If $\textrm{det}(\textrm{Jac}_{\Xi _n}) = 1$, then $\Xi _n$ is invertible.

### Proposition 9

($\chi _n$ is not invertible as a polynomial map) The polynomial map $\Xi _n$ is not invertible on $\mathbb {F}_2$.

### Proof

The determinant $\textrm{det}(\textrm{Jac}_{\Xi _n})$ contains a term $(-1)^{n+1} X_2 \cdot \textrm{det}(M_{n,1})$, where $M_{n,1}$ is the minor where the *n*th row and first column are deleted from the Jacobian. This factor does not cancel out, as can be seen from the shape of the matrix. $\square$

### Remark 3

The (in)famous Jacobian Conjecture states that a polynomial map is invertible if and only if the determinant of its Jacobian is invertible. Here, we used the easy-to-prove necessary condition.

### Definition 9

($\chi _n$ *on field extensions*) Let $\mathbb {F}_{2^k}$ be a field extension of $\mathbb {F}_2$ of degree *k*. We define $\chi ^{(k)}_n$ as the polynomial function indicated by the polynomial map $\Xi _n$ on the field $\mathbb {F}_{2^k}$.

Note that with this definition $\chi ^{(1)}_n = \chi _n$.

Since $\Xi _n$ is not invertible, while $\chi _n$ is invertible on $\mathbb {F}_2^n$, for odd *n*, it means that for some finite extension of $\mathbb {F}_2$, the polynomial function $\chi ^{(k)}_n$ is not invertible. This is due to the following result:

### Proposition 10

([31] Thm 4.2.1) Let *K* be an algebraically closed field. Let $F:K^n \rightarrow K^n$ be a polynomial function that is invertible. Then *F* is invertible as a polynomial map.

### Example 8

($\chi _3$ on $\mathbb {F}_4$) Consider the map

$$\begin{aligned} \chi ^{(2)}_3 :\mathbb {F}_4^3 \rightarrow \mathbb {F}_4^3,\ (x_0,x_1,x_2) \mapsto (x_0+(x_1+1)x_2, x_1+(x_2+1)x_0, x_2 + (x_0+1)x_1). \end{aligned}$$

Note that in $\mathbb {F}_4$ we have an element $\alpha$ with $\alpha ^2+\alpha +1 = 0$. Take the elements $(\alpha ,1,\alpha )$ and $(\alpha ,\alpha ,0)$. They all are mapped to $(\alpha ,0,1)$ under $\chi ^{(2)}_3$:

$$\begin{aligned} \chi ^{(2)}_3(\alpha ,1,\alpha )&= (\alpha + 0\cdot \alpha , 1 + (\alpha +1)\alpha , \alpha + (\alpha +1)\cdot 1) \\&= (\alpha , \alpha ^2+\alpha +1,\alpha +\alpha +1) = (\alpha ,0,1) \\ \chi ^{(2)}_3(\alpha ,\alpha ,0)&= (\alpha + (\alpha +1)\cdot 0, \alpha + \alpha , 0 + (\alpha +1)\alpha ) \\&= (\alpha , 0, \alpha ^2+\alpha ) = (\alpha ,0,1) \end{aligned}$$

It is therefore clear that $\chi ^{(2)}_3$ is not invertible.

The previous example generalizes for any odd $n>1$. Since $\chi _n$ is not invertible for even *n*, we immediately have $\chi ^{(k)}_n$ is not invertible either, for any $k>1$.

### Conjecture 1

($\chi _n$ is not invertible on any field extensions of $\mathbb {F}_2$) Let *n*, *k* be integers, both greater than 1 and *n* odd. Then $\chi ^{(k)}_n :\mathbb {F}_{2^k}^n \rightarrow \mathbb {F}_{2^k}^n$ is not invertible.

We conjecture the above, because we have found proofs for all even *k* and all *k* that are multiples of 3, as below. Note that we have $\mathbb {F}_{2^m} \subset \mathbb {F}_{2^l}$ if and only if $m \mid l$, hence we only have to check $k=2$ and $k=3$, as those examples work immediately in any extension of $\mathbb {F}_{2^2}$ or $\mathbb {F}_{2^3}$.

### Proof

(for $k=2$:) Let $n>1$ be odd. We will show a collision under $\chi ^{(2)}_n$. Let $\sigma _1 = (1,\alpha , 1, (1,0)^{\frac{n-3}{2}})$ and $\sigma _2 = (0,\alpha , \alpha ^2, (0,\alpha )^{\frac{n-3}{2}})$. Then $\chi ^{(2)}_n(\sigma _1) = \chi ^{(2)}_n(\sigma _2) = (\alpha , \alpha , 1, (0)^{n-3}).$ $\square$

### Proof

(for $k=3$:) Let $n>1$ be odd and $\alpha ^3+\alpha +1 = 0$. We will show a collision under $\chi ^{(3)}_n$. Let $\sigma _1 = (\alpha ^3,1,\alpha , (\alpha ^3,1)^{\frac{n-3}{2}})$ and $\sigma _2 = (\alpha ^6,\alpha ^4,\alpha ^6,(\alpha ^6,\alpha ^4)^{\frac{n-3}{2}})$. Then $\chi ^{(3)}_n(\sigma _1) = \chi ^{(3)}_n(\sigma _2) = (\alpha ^3,\alpha ^2,0,(\alpha ^3)^{n-3})$. $\square$

The remaining cases are open.^2^

It is interesting to see whether for different positive characteristics $\chi _n^{(k)}$ defined similarly is invertible and for which *k*, *n* this would be. It turns out, that $\chi _n^{(k)}$ is not invertible over characteristic *p* for any *n*, *k*.

### Proposition 11

($\chi _n$ is not invertible on any field of characteristic *p*) Let $p>2$ be a prime number. Let *n*, *k* be positive integers. Then $\chi ^{(k)}_n :\mathbb {F}_{p^k}^n \rightarrow \mathbb {F}_{p^k}^n$ is not invertible.

### Proof

Take $\sigma = 0^n$ and $\sigma ' = (p-2)^n$. Then for any index *i*, we have $\chi ^{(k)}_n(\sigma ')_i = \sigma '_i + (\sigma '_{i+1}+1)\sigma '_{i+2} = p-2 + (p-1)(p-2) = p\cdot (p-2) \equiv 0 \pmod {p}$. Thus $\chi ^{(k)}_n(\sigma ') = 0^n = \chi ^{(k)}_n(\sigma )$ for all *n*, *k*, *p*. $\square$

## Data Availability

This manuscript has no associated data.

## Appendix

### Proof of Proposition 5

We include here the proof of Proposition 5, since we have not been able to find one in the literature.

#### Proposition 12

[Differential probabilities for $\chi$ [8]] Let $n>1$ be an arbitrary odd integer. Let $a'\in \mathbb {F}_2^n$ be arbitrary. Then for any compatible $b'\in \mathbb {F}_2^n$ we have $\textrm{DP}_{\chi _n}(a',b') = 2^{-w(a')}$, where

$$\begin{aligned} w(a') = {\left\{ \begin{array}{ll} n-1 &{} \hbox {if } a' = 1^n; \\ \textrm{wt} (a') + r_{a'} &{} \hbox {else.} \end{array}\right. } \end{aligned}$$

where $r_{a'}$ is the number of 001-subsequences in $a'$.

#### Proof

We can express $b'$ in terms of $a'$ and *a* (here *a* is either of the two inputs that together have input difference $a'$) as follows (see [8] Sect. 6.9):

4 $$\begin{aligned} b' = \chi _n(a') + \underset{=: D_{a'}}{\underbrace{\begin{pmatrix} 0 &{}\quad a_2' &{}\quad a_1' &{}\quad 0 &{}\quad \cdots &{}\quad 0 &{}\quad 0 \\ 0 &{}\quad 0 &{}\quad a_3' &{}\quad a_2' &{}\quad \cdots &{}\quad 0 &{}\quad 0 \\ 0 &{}\quad 0 &{}\quad 0 &{}\quad a_4' &{}\quad \cdots &{}\quad 0 &{}\quad 0 \\ \vdots &{}\quad \vdots &{}\quad \vdots &{}\quad \vdots &{}\quad \ddots &{}\quad \vdots &{}\quad \vdots \\ 0 &{}\quad 0 &{}\quad 0 &{}\quad 0 &{}\quad \cdots &{}\quad a_{n-1}' &{}\quad a_{n-2}' \\ a_{n-1}' &{}\quad 0 &{}\quad 0 &{}\quad 0 &{}\quad \cdots &{}\quad 0 &{}\quad a_0' \\ a_1' &{}\quad a_0' &{}\quad 0 &{}\quad 0 &{}\quad \cdots &{}\quad 0 &{}\quad 0 \end{pmatrix}}} \cdot \begin{pmatrix} a_0 \\ a_1 \\ a_2 \\ \vdots \\ a_{n-3} \\ a_{n-2} \\ a_{n-1} \end{pmatrix} \end{aligned}$$

When the differential probability $\textrm{DP}(a',b') = 2^{-w(a')}$, then the dimension of the kernel of $D_{a'}$ is equal to $n-w(a')$. Therefore the rank of $D_{a'}$ will be equal to $w(a')$.

We will prove this by induction on the Hamming weight of $a'$, which we now denote as *k*:

$$\begin{aligned} {\mathcal {P}}(k) : \text {For all } n > k \text { and all } a'\in \mathbb {F}_2^n \text { such that } \textrm{wt} (a')=k, \text { we have } \textrm{rk} D_{a'} = w(a'). \end{aligned}$$

We start with the base case ${\mathcal {P}}(0)$.

Then for any $n > 0$, we have $\textrm{rk} D_{0^n} = 0$ since $D_{0^n}$ is the zero-matrix.

Indeed, $\textrm{DP}(0,b') = 2^0 = 1$ for all compatible $b'$ (of which there is only $b' = 0$).

The case ${\mathcal {P}}(1)$ is similar, as we may assume that $a_0' = 1$ and $a_i' = 0$ for $i \ne 0$. It is immediate that $\textrm{rk} D_{a'} = 2$. When $n \ge 3$, we have $r_{a'} = 1$ and $\textrm{wt} {a'} = 1$, hence $w(a') = 2 = \textrm{rk} D_{a'}$.

We now explore how we can extend an input difference $a'\in \mathbb {F}_2^{n-2}$ with $\textrm{wt} {a'} = k$ to an input difference $c'$ with $\textrm{wt} {c'} = k+1$. Consider the largest index *i* for which $a_i' = 1$.

By the shift-invariance of $\chi _n$ and the properties of differential probability for linear maps, we can assume that $i = n-3$.

We can concatenate one of the following to $a'$:

1. 10;
2. 01;
3. $$0^\ell 1(0)$$.

With (0) we denote that we concatenate another zero if $\ell$ is even, and do not concatenate it if $\ell$ is odd. Note that this lists all possibilities to extend an input difference $a'$ to a longer sequence $c'$ with $\textrm{wt} {c'} = \textrm{wt} {a'}+1$.

Consider some $a'\in \mathbb {F}_2^{n-2}$ such that $\textrm{wt} {a'}=k$ with $a_{n-3}' =1$. We will show that ${\mathcal {P}}(k+1)$ is true, for case 1.

1. Let $c' = a'\Vert 10$ and $d' = a'\Vert 00$. Both $D_{c'}$ and $D_{d'}$ are $n\times n$ matrices. By the induction hypothesis, we know that $\textrm{rk} D_{d'} = \textrm{wt} {d'} + r_{d'}$. We make a case distinction:
  - Either $d'$ starts with $0^l1$ for $l>1$;
  - or $d'$ starts with 01;
  - or $d'$ starts with 1.
  We now assume each of these cases separately.
  - We have $\textrm{wt} {c'} = \textrm{wt} {d'}+1$ and $r_{c'} = r_{d'}$. Thus, we have to show that $\textrm{rk} D_{c'} = \textrm{rk} D_{d'} + 1$. For that, we consider the following submatrix of $D_{a'}$:
    $$\begin{aligned} \textrm{sub}.D_{a'} := \begin{matrix} a_{n-3}' &{}\quad a_{n-4}' &{}\quad 0 &{}\quad 0 \\ 0 &{}\quad a_{n-2}' &{}\quad a_{n-3}' &{}\quad 0 \\ 0 &{}\quad 0 &{}\quad a_{n-1}' &{}\quad a_{n-2}' \\ 0 &{}\quad 0 &{}\quad 0 &{}\quad a_0' \end{matrix} \end{aligned}$$
    We then note that all other coordinates of $D_{a'}$ do not change when we go from $D_{d'}$ to $D_{c'}$. We have:
    **Table:**
    	$a_{n-4}'$	$a_{n-3}'$	$a_{n-2}'$	$a_{n-1}'$	$a_0'$
    $c':$	?	1	1	0	0
    $d':$	?	1	0	0	0
    The given columns are extended upwards and downwards with 0s in the matrix $D_{a'}$. The same holds for the rows, that are extended leftwards with 0s, except for the last one, which has $a_{n-1}'$ in its first position. There we, thus, have
    $$\begin{aligned} \textrm{sub}.D_{d'} := \begin{matrix} 1 &{}\quad a_{n-4}' &{}\quad 0 &{}\quad 0 \\ 0 &{}\quad 0 &{}\quad 1 &{}\quad 0 \\ 0 &{}\quad 0 &{}\quad 0 &{}\quad 0 \\ 0 &{}\quad 0 &{}\quad 0 &{}\quad 0 \end{matrix} \qquad \qquad \textrm{sub}.D_{c'} := \begin{matrix} 1 &{}\quad a_{n-4}' &{}\quad 0 &{}\quad 0 \\ 0 &{}\quad 1 &{}\quad 1 &{}\quad 0 \\ 0 &{}\quad 0 &{}\quad 0 &{}\quad 1 \\ 0 &{}\quad 0 &{}\quad 0 &{}\quad 0 \end{matrix} \end{aligned}$$
    In this forelying case, $a_{n-1}'=0$, hence this submatrix is independent on the other blocks in $D_{a'}$. It is immediately clear by looking at the first three rows of the submatrices, that $\textrm{rk} D_{c'} = \textrm{rk} D_{d'} + 1$.
  - This case is identical to 1a.
  - We have $\textrm{wt} {c'} = \textrm{wt} {d'}+1$ and $r_{c'} = r_{d'}-1$. Thus, we have to show that $\textrm{rk} D_{c'} = \textrm{rk} D_{d'}$. We have:
    **Table:**
    	$a_{n-4}'$	$a_{n-3}'$	$a_{n-2}'$	$a_{n-1}'$	$a_0'$
    $c':$	?	1	1	0	1
    $d':$	?	1	0	0	1
    and thus
    $$\begin{aligned} \textrm{sub}.D_{d'} := \begin{matrix} 1 &{} a_{n-4}' &{} 0 &{} 0 \\ 0 &{} 0 &{} 1 &{} 0 \\ 0 &{} 0 &{} 0 &{} 0 \\ 0 &{} 0 &{} 0 &{} 1 \end{matrix} \qquad \qquad {sub}. D_{c'} := \begin{matrix} 1 &{} a_{n-4}' &{} 0 &{} 0 \\ 0 &{} 1 &{} 1 &{} 0 \\ 0 &{} 0 &{} 0 &{} 1 \\ 0 &{} 0 &{} 0 &{} 1 \end{matrix} \end{aligned}$$
    wherefrom it is clear that $\textrm{rk} D_{c'} = \textrm{rk} D_{d'}$.
  Therefore, case 1. has been shown.
2. The other two cases go in a similar fashion, by noting the two preceding and succeeding bits down and choosing the right submatrices. In the full version of this paper, see [29], we have included cases 2 and 3.
3. See 2.

By the above case distinction, we have proven half of the proposition by means of induction.

For the other half, namely that $w(a') = n-1$, when $a' = 1^n$, we just need to show that the rank of $D_{a'} = n-1$. This follows by doing elementary row reductions. The echelon form will consist of an $(n-1)\times (n-1)$ identity matrix $I_{n-1}$, with an $(n-1)\times 1$ all-one column to the right of it, and an all-zero row below all this. $\square$

### Actual sparsity and degree

Here, we list the actual numbers for the minimum sparsity, maximum sparsity, minimum degree and maximum degree for univariate representations of $\chi _n$ for several *n*.

Below those, we also give tables for the exact different univariate representations for $\chi _n$.

**Table 6:** The true bounds on sparsity and degree for univariate expressions for $\chi _n$

	Min. sparsity	Max. sparsity	Min. degree	Max. degree
$n=3$	1	3	6	6
$n=5$	5	9	18	24
$n=7$	11	19	64	96
$n=9$	15	33	258	384

In the Tables 7, 8, and 9, for $\chi _3,\chi _5,\chi _7$, we have $\sigma \in S_n =\textrm{Sym}(\{0,\ldots ,n-1\})$.

**Table 7:** Univariate representations of $\chi _3$

Normal element	Iso. ($\sigma$)	Resulting polynomial
$\beta ^3 + \beta ^2 + 1 = 0$	$\textrm{id}$	$t^6$
	$(1\ 2)$	$t^6 + t^4 + t^2$

**Table 8:** Univariate representations of $\chi _5$

Normal element	Iso. ($\sigma$)	Resulting polynomial
$\beta ^5 + \beta ^4 + \beta ^2 + \beta + 1 = 0$	$\textrm{id}$	$t^{20} + t^{12} + t^8 + t^6 + t^5 + t^4 + t^3$
	$(1\ 2\ 4\ 3)$	$t^{18} + t^{17} + t^{10} + t^6 + t^5$
	$(1\ 3\ 4\ 2)$	$t^{20} + t^{16} + t^{12} + t^{10} + t^5 + t^4 + t^3 + t^2 + t$
	$(1\ 4)(2\ 3)$	$t^{24} + t^{17} + t^9 + t^8 + t^5 + t^4 + t^3$
$\beta ^5 + \beta ^4 + \beta ^3 + \beta + 1 = 0$	$\textrm{id}$	$t^{24} + t^{18} + t^{17} + t^{16} + t^4 + t^3 + t$
	$(1\ 2\ 4\ 3)$	$t^{24} + t^{20} + t^{16} + t^{10} + t^9 + t^8 + t^5 + t^4 + t^2$
	$(1\ 3\ 4\ 2)$	$t^{20} + t^{18} + t^{17} + t^{10} + t^9 + t^8 + t^2$
	$(1\ 4)(2\ 3)$	$t^{24} + t^{20} + t^{12} + t^6 + t^2$
$\beta ^5 + \beta ^4 + \beta ^3 + \beta ^2 + 1 = 0$	$\textrm{id}$	$t^{24} + t^{10} + t^9 + t^6 + t^5 + t^2 + t$
	$(1\ 2\ 4\ 3)$	$t^{20} + t^{17} + t^{12} + t^8 + t^4 + t^3 + t$
	$(1\ 3\ 4\ 2)$	$t^{24} + t^9 + t^8 + t^6 + t^4 + t^3 + t$
	$(1\ 4)(2\ 3)$	$t^{18} + t^{17} + t^{16} + t^{10} + t^9 + t^6 + t^4 + t^2 + t$

In Table 9, we write for the normal element a set of non-negative integers. These denote the exponents of the monomials that have coefficient 1 in the defining polynomial of the normal element. For instance, $\{7,6,5,2,0\}$ denotes $\beta ^7 + \beta ^6 + \beta ^5 + \beta ^2 + 1 = 0$.

Similarly, we write a tuple of non-negative integers for the resulting polynomials.

**Table 9:** Univariate representations of $\chi _7$

Normal element	Iso. ($\sigma$)	Resulting polynomial
$\{7, 6, 5, 2, 0\}$	$\textrm{id}$	(96, 80, 68, 48, 40, 34, 33, 32, 24, 18, 16, 12, 9, 8, 2)
	$(1\ 2\ 4)(3\ 6\ 5)$	(80, 66, 48, 40, 33, 32, 24, 20, 12, 10, 9, 4, 1)
	$(1\ 3\ 2\ 6\ 4\ 5)$	(96, 68, 66, 65, 48, 36, 34, 32, 24, 18, 9, 8, 4)
	$(1\ 4\ 2)(3\ 5\ 6)$	(96, 72, 65, 64, 36, 32, 18, 17, 16, 10, 9, 8, 6, 4, 3)
	$(1\ 5\ 4\ 6\ 2\ 3)$	(80, 68, 40, 33, 24, 20, 12, 10, 6, 5, 4, 2, 1)
	$(1\ 6)(2\ 5)(3\ 4)$	(96, 72, 66, 65, 64, 36, 18, 17, 16, 10, 8, 6, 5, 4, 3)
$\{7,6,4,2,0\}$	$\textrm{id}$	(96, 80, 72, 68, 66, 64, 36, 34, 32, 20, 9, 8, 6, 3, 1)
	$(1\ 2\ 4)(3\ 6\ 5)$	(96, 80, 40, 34, 32, 24, 20, 18, 16, 10, 8, 6, 2)
	$(1\ 3\ 2\ 6\ 4\ 5)$	(96, 80, 48, 32, 24, 18, 17, 9, 6, 5, 3)
	$(1\ 4\ 2)(3\ 5\ 6)$	(96, 72, 68, 66, 65, 64, 36, 24, 20, 12, 8, 4, 3)
	$(1\ 5\ 4\ 6\ 2\ 3)$	(64, 48, 40, 20, 17, 12, 10, 9, 8, 5, 3)
	$(1\ 6)(2\ 5)(3\ 4)$	(72, 68, 64, 48, 36, 34, 33, 24, 20, 18, 6, 5, 1)
$\{7,6,4,1,0\}$	$\textrm{id}$	(96, 80, 66, 48, 40, 36, 33, 24, 18, 12, 10, 6, 5, 4, 2
	$(1\ 2\ 4)(3\ 6\ 5)$	(96, 68, 66, 65, 48, 40, 36, 34, 32, 24, 20, 18, 12, 4, 3, 2, 1)
	$(1\ 3\ 2\ 6\ 4\ 5)$	(80, 72, 68, 65, 34, 33, 10, 8, 5, 3, 2)
	$(1\ 4\ 2)(3\ 5\ 6)$	(66, 64, 40, 34, 32, 20, 18, 12, 9, 6, 5, 4, 1)
	$(1\ 5\ 4\ 6\ 2\ 3)$	(96, 65, 48, 34, 33, 24, 20, 18, 17, 12, 10, 9, 6, 4, 2)
	$(1\ 6)(2\ 5)(3\ 4)$	(96, 80, 66, 65, 48, 40, 34, 32, 20, 17, 16, 10, 8, 6, 5, 4, 3, 2, 1)
$\{7,6,3,1,0\}$	$\textrm{id}$	(80, 64, 40, 34, 33, 32, 24, 20, 18, 12, 10, 8, 4, 3, 2)
	$(1\ 2\ 4)(3\ 6\ 5)$	(80, 68, 48, 36, 33, 24, 18, 16, 12, 10, 6, 4, 3)
	$(1\ 3\ 2\ 6\ 4\ 5)$	(68, 65, 64, 34, 33, 32, 24, 18, 17, 10, 5, 4, 1)
	$(1\ 4\ 2)(3\ 5\ 6)$	(96, 72, 68, 64, 40, 20, 18, 16, 9, 8, 6, 5, 2)
	$(1\ 5\ 4\ 6\ 2\ 3)$	(80, 65, 64, 40, 34, 24, 18, 16, 12, 9, 8, 6, 5, 4, 3, 2, 1)
	$(1\ 6)(2\ 5)(3\ 4)$	(96, 80, 66, 65, 64, 33, 32, 24, 18, 17, 16, 10, 8, 5, 1)
$\{7,6,0\}$	$\textrm{id}$	(96, 68, 65, 64, 48, 36, 34, 33, 32, 20, 16, 12, 10, 6, 5, 4, 3, 2, 1)
	$(1\ 2\ 4)(3\ 6\ 5)$	(72, 68, 66, 65, 64, 48, 40, 34, 32, 20, 18, 17, 10, 8, 6, 5, 4, 3, 2)
	$(1\ 3\ 2\ 6\ 4\ 5)$	(80, 68, 48, 40, 36, 33, 32, 24, 20, 17, 16, 8, 5, 3, 2)
	$(1\ 4\ 2)(3\ 5\ 6)$	(96, 80, 68, 66, 65, 33, 32, 20, 18, 17, 12, 5, 4)
	$(1\ 5\ 4\ 6\ 2\ 3)$	(96, 72, 66, 65, 64, 36, 34, 33, 32, 24, 20, 17, 10, 9, 5, 3, 2)
	$(1\ 6)(2\ 5)(3\ 4)$	(96, 80, 48, 40, 36, 34, 33, 32, 24, 18, 16, 12, 10, 6, 3)
$\{7,6,5,3,2,1,0\}$	$\textrm{id}$	(80, 64, 48, 40, 36, 32, 24, 20, 16, 12, 10, 8, 5, 4, 3)
	$(1\ 2\ 4)(3\ 6\ 5)$	(68, 65, 36, 34, 33, 24, 20, 18, 17, 12, 9, 6, 5, 4, 3)
	$(1\ 3\ 2\ 6\ 4\ 5)$	(80, 66, 40, 36, 34, 33, 18, 17, 16, 10, 5, 4, 3, 2, 1)
	$(1\ 4\ 2)(3\ 5\ 6)$	(72, 68, 66, 40, 33, 20, 17, 16, 12, 10, 9, 5, 1)
	$(1\ 5\ 4\ 6\ 2\ 3)$	(96, 80, 72, 68, 66, 65, 64, 34, 18, 17, 16, 12, 10, 9, 6, 4, 3, 2, 1)
	$(1\ 6)(2\ 5)(3\ 4)$	(96, 80, 66, 65, 64, 40, 34, 33, 32, 24, 8, 5, 3)
$\{7,6,5,4,2,1,0\}$	$\textrm{id}$	(66, 64, 48, 40, 36, 34, 33, 18, 16, 12, 6, 5, 4)
	$(1\ 2\ 4)(3\ 6\ 5)$	(96, 80, 66, 65, 48, 34, 20, 18, 12, 10, 9, 8, 4, 2, 1)
	$(1\ 3\ 2\ 6\ 4\ 5)$	(80, 72, 66, 65, 36, 34, 32, 24, 17, 8, 5, 3, 1)
	$(1\ 4\ 2)(3\ 5\ 6)$	(96, 68, 66, 64, 34, 32, 20, 17, 12, 10, 9, 8, 6)
	$(1\ 5\ 4\ 6\ 2\ 3)$	(96, 68, 48, 40, 33, 24, 17, 16, 10, 9, 6, 5, 2)
	$(1\ 6)(2\ 5)(3\ 4)$	(96, 66, 48, 40, 34, 32, 20, 18, 17, 10, 8, 3, 2)

## Footnotes

1 Between submission and publication of this paper, another proof has been found in [16].

2 Between submission and publication of this paper, this conjecture has been studied in two preprints [25] and [16].
